# Pseudo Effects: How Method Biases Can Produce Spurious Findings About Close Relationships

**DOI:** 10.1177/09567976251370262

**Published:** 2025-09-10

**Authors:** Samantha Joel, John K. Sakaluk, James J. Kim, Devinder Khera, Helena Yuchen Qin, Sarah C. E. Stanton

**Affiliations:** 1 Department of Psychology, Western University; 2 Department of Psychology, University of Edinburgh

**Keywords:** interpersonal relationships, method biases, construct validity, sentiment override, relationship quality, measurement

## Abstract

Research on interpersonal relationships frequently relies on accurate self-reporting across various relationship facets (e.g., conflict, trust, appreciation). Yet shared method biases—which may greatly inflate associations between measures—are rarely accounted for during measurement validation or hypothesis testing. To examine how method biases can affect relationship research, we embarked on the ironic exploration of a new construct—*Pseudo*—comprised of irrelevant relationship evaluations (e.g., “My relationship has very good Saturn”). Pseudo was moderately associated with common relationship measures (e.g., satisfaction, commitment) and predicted those measures 3 weeks later. Results of a dyadic longitudinal study suggested that Pseudo taps into method biases, particularly *sentiment override* (i.e., people’s tendency to project their global relationship sentiments onto every relationship evaluation). We conclude that psychometric standards must be sufficiently rigorous to distinguish genuine constructs and associations from methodological artifacts that can otherwise pose a serious validity threat.

Psychology is facing renewed calls to reconsider its measurement practices (e.g., Elson et al., 2023; Flake & Fried, 2020; Vazire et al., 2022). Growing metascientific evidence suggests that standard scale-validation techniques—such as those used to probe a measure’s reliability (McNeish, 2018), factor structure (e.g., Hussey & Hughes, 2020; Maul, 2017; Sakaluk & Short, 2017), and predictive utility (Westfall & Yarkoni, 2016)—do not offer sufficiently strong tests of a measure’s construct validity. For example, one pair of studies suggests that factor analysis can be used to validate constructs that lack theoretical substance (Maul, 2017). Further, simulation studies suggest that psychology’s standard approach to statistical control can produce substantially inflated Type 1 error rates (Westfall & Yarkoni, 2016).

One measurement challenge particularly worthy of renewed attention is *common method variance*: variance that is attributable to how the measures were collected rather than to the constructs they are intended to represent (Podsakoff et al., 2024). Common method variance can artificially inflate associations between measures collected with the same method (e.g., self-report). For example, a researcher may find an association between self-report measures of affection and sexual desire and infer that people experience higher desire when their partners show greater affection. Yet participants may rate those measures similarly not because the underlying constructs (affection and desire) are related, but because of a motivation to present the relationship favorably (social desirability; Nederhof, 1985), a tendency to rate most items positively (acquiescence bias; e.g., Van Herk et al., 2004), or because their current mood shaped their responses (see Podsakoff et al., 2003, for other examples).

In the present research, we consider the consequences of shared-method biases when psychologists attempt to study close relationships—one of the broadest areas of psychological inquiry, spanning social, developmental, and clinical areas of investigation. This research area has generated an enormous set of constructs (see Finkel et al., 2017, and Reis, 2007, for reviews), each accompanied by one or more self-report measures (Joel et al., 2020) developed with psychometric techniques. A systematic review found that most close relationship studies (83%) rely on self-report as their only method of measurement (Williamson et al., 2022). These studies are thus susceptible to the sorts of method biases that have been uncovered in other self-report-heavy fields, such as personality (McCrae, 2018), organizational behavior (Donaldson & Grant-Vallone, 2002), and marketing (MacKenzie & Podsakoff, 2012). Yet relationship science, unlike these neighboring fields, has yet to seriously grapple with the problem of method biases (for discussion, see Joel et al., 2025; Orth, 2013; and Wang & Eastwick, 2020).

Beyond domain-general method biases (e.g., Podsakoff et al., 2003), psychologists studying close relationships may need to contend with a domain-specific source of method bias: *sentiment override* (Weiss, 1980). When people feel positively toward close others, those global positive sentiments can greatly shape their perceptions of more specific aspects of their relationships (e.g., Fincham et al., 1995; Waldinger & Schulz, 2006). For example, people in satisfying romantic relationships tend to feel highly understood by their partners, regardless of how well their partners actually know them (Hinnekens et al., 2020). To date, sentiment override has primarily been a phenomenon of theoretical interest (e.g., Fincham et al., 1995; Weiss, 1980). But sentiment override may also pose methodological challenges: Measures of specific relationship experiences (e.g., “My partner was affectionate toward me today”) inadvertently capture global evaluations as well (“I have a good romantic partner”). This shared-method effect may even leave researchers at risk of developing tautological models. For example, a study might appear to show that affection predicts sexual desire, when it actually shows that satisfaction (embedded within the affection items) predicts satisfaction (embedded within the desire items).

## The present research

To investigate the extent to which scholars of interpersonal processes should be concerned about method biases, we developed a conceptually irrelevant scale (akin to that of Maul, 2017)—labelled *Pseudo*—composed of items that are syntactically evaluative of romantic relationships (e.g., “My partner and I have excellent taro”) These items are relationship specific, and should thus be well suited to capture sentiment-override processes. However, they do not tap into any substantive aspect of the relationship, so they do not represent a useful new construct (at best, they represent a useful old construct: global relationship sentiments). We therefore used Pseudo as a stress test of standard construct-validation practices in the field. The extent to which Pseudo can predict relationship outcomes would reveal how readily new measures can produce evidence of predictive validity in the absence of novel theoretical substance.

## Research Transparency Statement

### General disclosures

**Conflicts of interest:** All authors declare no conflicts of interest. **Funding:** This work was supported by Canada Social Sciences and Humanities Research Council (SSHRC) Insight Grant No. 435-2019-0115 and by UK Economic and Social Research Council (ESRC) New Investigator Grant No. ES/S005749/1. **Artificial intelligence:** No artificial-intelligence-assisted technologies were used in this research or the creation of this article. **Ethics:** Study 1 received approval from the Western University Non-Medical Research Ethics Board (Project ID No. 119231), and Study 2 received approval from the University of Edinburgh PPLS Research Ethics Committee (Ref. No. 422-2122/1). **Computational reproducibility:** The computational reproducibility of the results in the main article (but not the Supplemental Material) has been independently confirmed by the journal’s STAR team. **Open Science Framework (OSF):** To ensure long-term preservation, we registered all OSF files at https://osf.io/8zh9d.

### Pilot study disclosures

**Preregistration:** The pilot study was not preregistered. **Materials:** All materials are publicly available (https://osf.io/vjnrp/). **Data:** All primary data are publicly available (https://osf.io/vjnrp/). **Analysis scripts:** All analysis scripts are publicly available (https://osf.io/vjnrp/).

### Study 1a disclosures

**Preregistration:** Study 1a was not preregistered. **Materials:** All materials are publicly available (https://osf.io/2tqke/). **Data:** All primary data are publicly available (https://osf.io/2tqke/). **Analysis scripts:** All analysis scripts are publicly available (https://osf.io/2tqke/).

### Study 1b disclosures

**Preregistration:** Study 1b was not preregistered. **Materials:** All materials are publicly available (https://osf.io/knca8/). **Data:** All primary data are publicly available (https://osf.io/knca8/). **Analysis scripts:** All analysis scripts are publicly available (https://osf.io/knca8/).

### Study 2 disclosures

**Preregistration:** The hypotheses and analysis plan regarding the baseline data were preregistered on March 27, 2023: https://osf.io/jpc6z. The hypotheses and analysis plan regarding the longitudinal data were preregistered on September 1, 2023: https://osf.io/mc52r. In each case, the preregistration was submitted after data collection was complete, but before the first and second authors had access to the data. Data were collected between October 2022 and February 2023. There were minor deviations from the preregistered plan, which are reported in the Supplemental Material. **Materials:** All materials are publicly available (https://osf.io/sprgj/). **Data:** Data are not publicly available because of confidentiality concerns. Data are available upon request from S. C. E. Stanton (Sarah.Stanton@ed.ac.uk). **Analysis scripts:** All analysis scripts are available (https://osf.io/sprgj/).

## Study 1a

We constructed a 10-item measure of irrelevant relationship evaluations and administered it in the context of a weekly experience study. Participants completed standard state measures of satisfaction, commitment, appreciation, and trust, each week for 4 weeks. We evaluated Pseudo as a relationship construct using several standard scale-validation approaches. Specifically, we tested whether Pseudo (a) had a defensible factor structure, (b) had defensible cross-sectional associations with established relationship constructs, and (c) had defensible longitudinal associations with these constructs. Positive coefficients were taken as spurious evidence of Pseudo’s validity as a substantive new relationship construct. Materials, analysis scripts, and primary data are all publicly available (https://osf.io/2tqke/).

### Method

#### Participants and procedure

We recruited 495 participants from Western University’s mass email recruitment LISTSERV and from social media (e.g., Reddit, Twitter, Instagram). We excluded 18 participants who were not in long-term romantic relationships. The final sample consisted of 477 participants (149 men, 315 women, 6 nonbinary, 6 other, and 1 preferred not to say) with a mean age of 25 (*SD* = 5.37 years, age range = 18–59 years). This final sample size is well above threshold for a simple model with typical communalities for psychological factor-analysis models (see MacCallum et al., 1999). Participants were primarily heterosexual (*n* = 358; 75.1%), White (*n* = 264; 55.3%), and cisgender (*n* = 457; 95.8%). See Table S2 in the Supplemental Material for detailed participant demographics.

Participants completed a baseline questionnaire in Week 1 and a slightly shorter questionnaire each week for the subsequent 3 weeks. Participants completed an average of three out of four surveys.^1^

#### Measures

##### Relationship outcomes

Satisfaction (e.g., “At this moment, how satisfied are you with your relationship?”; Week 1: α = .94) and commitment (e.g., “At this moment, how committed are you with your relationship?”; Week 1: α = .95) were each measured on each of the 4 weeks using 3 items adapted from the Perceived Relationship Quality Component (PRQC) Inventory (Fletcher et al., 2000). Appreciation was measured each week with three items adapted from Gordon and Chen (2010; e.g., “I feel very lucky to have my partner in my life,” Week 1: α = .88). Trust was measured each week with three items adapted from Rempel et al. (1985; e.g., “I can count on my partner to be concerned about my welfare,” Week 1: α = .87).

##### Pseudo items

On the Week 2 survey only, participants were shown the following prompt: “These next questions may seem a bit strange. It’s okay if you’re not sure what they mean. Please answer them anyway to the best of your ability.” They were then presented with 10 pseudoevaluative relationship items (e.g., “My partner and I have excellent taro”) on a scale ranging from 1, *strongly disagree*, to 7, *strongly agree* (α = .95). Each item followed the syntax of a common relationship measure (e.g., “My partner and I have excellent _____”), with the target noun (e.g., *love*, *communication*, *passion*) replaced with a plant or mineral (e.g., *daffodil*, *snowdrop*, *umbra*, *terra*).

### Results

We first examined the new scale’s factor structure using a separate sample of participants in relationships (pilot study; *N* = 331). An exploratory factor-analysis model with one factor accounted for 58% of the variance, and all 10 items loaded highly onto the single factor (all loadings > .70), suggesting that this one-factor solution was well suited to the data. (See Table 1.) These pilot data are described in full in the Supplemental Material available online.

**Table 1.** Piloted Pseudo Items

Scale item	Loading	*M*	*SD*
My relationship has very good Saturn	.71	4.76	1.25
The daffodil in my relationship is close to ideal	.72	4.74	1.31
The midnight in my relationship continues to improve over time	.73	4.84	1.25
I love the snowdrop that I get from my relationship	.79	4.84	1.34
I enjoy the dolomite that I share with my partner	.75	4.83	1.34
My partner and I have excellent taro	.74	4.91	1.38
I appreciate the umbra that my relationship brings	.81	4.94	1.27
I love the fuchsine that my partner gives me	.74	4.86	1.33
I treasure the reseda in my relationship	.82	4.83	1.22
My partner and I share incredible terra	.80	5.07	1.33

Using the weekly experience sample, we tested a confirmatory factor-analytic model in which a single latent factor predicted all 10 Pseudo items, estimated using the *lavaan* package in R, with robust maximum likelihood estimation and standardized loadings. This model yielded acceptable fit according to standard fit criteria (Hu & Bentler, 1999), χ^2^(35) = 105.488, *p* < .001, comparative fit index (CFI) = .978, root-mean-square error of approximation (RMSEA) = .070, standardized root-mean-square residual (SRMR) = .023. All 10 items loaded well onto the single factor (all *bs* > 0.70).

Correlations between Pseudo and established relationship constructs measured at the same time point (Week 2) can be seen in Table 2. Pseudo was moderately to highly correlated with each of the other constructs, all *rs* from .43 to .58. These correlations suggest that these relationship constructs are in part tapping into sources of method biases, such as sentiment override. The other relationship measures were highly correlated with each other (all *r*s = .60–.80).

**Table 2.** Associations Between Pseudo and Relevant Relationship Constructs in Study 1

Variable	Satisfaction	Commitment	Appreciation	Trust
Pseudo	.584	.437	.431	.430
Satisfaction		.771	.763	.734
Commitment			.696	.599
Appreciation				.798

### Does Pseudo predict relationship outcomes from week to week?

Did people who reported higher Pseudo at baseline experience better relationship outcomes from week to week? We constructed two-level multilevel models (time points nested within participants) using the *lme4* package in R (Bates et al., 2015). Data were structured at the weekly level. Pseudo (level 2, grand-mean-centered) was entered as the sole predictor in each of four models predicting each weekly relationship construct (level 1, uncentered).

Indeed, Pseudo predicted better outcomes on all four relationship constructs. People who reported higher Pseudo experienced higher satisfaction, *b* = 0.521, *SE* = .040, *p* < .001; commitment, *b* = 0.349, *SE* = .037, *p* < .001; appreciation, *b* = 0.438, *SE* = .039, *p* < .001; and trust, *b* = 0.431, *SE* = .038, *p* < .001, from week to week. All results also held when the three other weekly relationship constructs were included as covariates (level 1, group-mean-centered; see Table 3). For example, people with higher Pseudo at baseline were more satisfied in their relationship on a given week regardless of whether they felt more or less committed, appreciative, or trusting that week than they usually did.

**Table 3.** Pseudo Predicts Weekly Relationship Outcomes Above and Beyond Weekly Covariates

Predictor	Weekly satisfaction			Weekly commitment			Weekly appreciation			Weekly trust		
	*b*	*SE*	*p*	*b*	*SE*	*p*	*b*	*SE*	*p*	*b*	*SE*	*p*
Pseudo	0.520	.040	< .001	0.347	.037	< .001	0.439	.039	< .001	0.430	.038	< .001
Weekly satisfaction				0.405	.029	< .001	0.201	.028	< .001	0.141	.031	< .001
Weekly commitment	0.403	.029	< .001				0.179	.028	< .001	0.033	.032	.29
Weekly appreciation	0.235	.033	< .001	0.211	.033	< .001				0.548	.030	< .001
Weekly trust	0.138	.031	< .001	0.033	.031	.288	0.458	.025	< .001			

### Does Pseudo predict change in relationship variables over time?

We next examined whether Pseudo could predict change in relationship variables from the beginning to the end of the study. We conducted a series of multiple regression analyses in which Pseudo (collected at Week 2, grand-mean-centered) and each of the four established relationship constructs at baseline (uncentered) were entered as simultaneous predictors, predicting that same relationship construct at Week 4 (uncentered). We tested a version of these models in which Week 1 was treated as baseline (the earliest week of data collected), as well as a version in which Week 2 was treated as baseline (the same week in which Pseudo was measured).

Results are shown in Table 4. Indeed, Week 2 Pseudo significantly predicted satisfaction, commitment, appreciation, and trust at Week 4, controlling for the equivalent measure at Week 1. Pseudo also significantly predicted appreciation and trust at Week 4, and marginally predicted commitment at Week 4, controlling for the equivalent measure at Week 2. On the basis of the current statistical conventions of the field, these results suggest that people with higher Pseudo generally experienced more positive changes in relationship outcomes over a period of several weeks than those with lower Pseudo.

**Table 4.** Pseudo Predicts Change in Relationship Variables Using Multiple Regression

Predictor	Baseline is Week 1			Baseline is Week 2		
	Week 4 relationship outcome					
	*b*	*SE*	*p*	*b*	*SE*	*p*
Model 1						
Pseudo	0.205	.052	< .001	0.068	.050	.176
Baseline satisfaction	0.709	.055	< .001	0.796	.049	< .001
Model 2						
Pseudo	0.186	.047	< .001	0.081	.043	.060
Baseline commitment	0.612	.051	< .001	0.770	.047	< .001
Model 3						
Pseudo	0.233	.048	< .001	0.181	.043	< .001
Baseline appreciation	0.591	.052	< .001	0.682	.044	< .001
Model 4						
Pseudo	0.244	.046	< .001	0.198	.044	< .001
Baseline trust	0.627	.049	< .001	0.690	.045	< .001

## Study 1b

How is it that a seemingly meaningless scale can pass standard validity tests? To find out, we next qualitatively probed participants’ interpretations of the Pseudo items. Best practices for scale validation involve gathering *process evidence*: qualitative data on whether participants are interpreting the scale as intended (Standards for Educational and Psychological Testing; see American Educational Research Association et al., 2014). However, qualitative scale-validation methods are uncommon within relationship science. Indeed, the authors reviewed the validity evidence for 44 common relationship measures (see the Supplemental Material) and found that only one scale included qualitative data about what participants think the measure means (the Inclusion of Other in Self Scale; Aron et al., 1992). The goal of the current study was to gather process evidence for Pseudo. What kind of meaning, if any, do participants tend to project onto the Pseudo items?

### Method

#### Participants

Participants were recruited on Western University’s mass email recruitment LISTSERV in June 2024. The final sample consisted of 81 participants (10 men, 37 women, 1 nonbinary, 33 who did not specify their gender), with a mean age of 24.77 (*SD* = 6.11 years, range = 18–46). The participants were primarily heterosexual (*n* = 31; 38.27%), White (*n* = 27; 33.33%), and in nonmarried committed relationships (*n* = 31; 38.27%).

#### Pseudo items

Participants were shown the following prompt: “These next questions may seem a bit strange. It’s okay if you’re not sure what they mean. Please answer them anyway to the best of your ability.” They were then asked to rate each of three Pseudo items: “I love the snowdrop that I get from my relationship,” “I appreciate the umbra that my relationship brings,” and “I treasure the reseda in my relationship.” These three Pseudo items were chosen because they had the highest loadings in previous samples. The order of the Pseudo items was randomized.

#### Open-ended reasoning

After each Pseudo item, participants were then presented with three open-ended questions: a category-selection probe, a specific probe, and a comprehension probe (Behr et al., 2017). The *category-selection probe* asked participants to explain why they chose their selected value rather than an adjacent value. Next, the *specific probe* asked participants to explain their reasoning in more detail: “What experiences were you thinking of when you rated this statement?” Finally, the *comprehension probe* asked participants to describe their understanding of the question: “In your own words, what do you think this statement means?”

More detailed methodology (e.g., sampling procedure, participant demographics, and wording of the prompts) and results (e.g., the coding scheme and the qualitative themes) are available in the Supplemental Material. Materials and primary data are publicly available (https://osf.io/knca8/).

### Study 1b results

The first and fifth authors first read the open-ended responses for general themes, from which they generated a detailed coding scheme. They next coded the open-ended responses according to this scheme, and codes were finalized by the fifth author. Interrater reliability between coders was assessed using unweighted Cohen’s kappa, with values ranging from 0.76 to 0.95.

#### Theme 1: confusion

Most participants (90.12%) expressed confusion or uncertainty about the meaning of at least one of the items. In 67.24% of cases in which participants expressed confusion, they also gave a neutral response to the item (e.g., “I chose 4 ‘neither agree nor disagree’ because I don’t know what the word reseda means”).

#### Theme 2: relationship interpretations

Despite the unclear meaning of the items, many participants tried to rate them by drawing on information about their own romantic relationships. A total of 88.89% of participants referenced their own relationships in response to at least one item, and many of these responses (41.46%) drew on global, usually positive feelings about the relationship. For example, when asked to justify a rating to the snowdrop item, a participant wrote, “I love the person I am in a relationship with. 7 was the highest number I could choose to indicate how much I love them.” In response to the umbra item, another wrote, “I wasn’t sure what umbra meant. But I appreciate everything that comes with my relationship, so I clicked ‘agree.’”

In addition, many of the responses (68.29%) drew on specific, concrete relationship qualities, often including a multitude of them simultaneously. For example, when asked what they were thinking about when completing the snowdrop item, a participant wrote, “I was thinking of the consistent support, affection, and small acts of kindness I receive in my relationship, which make me feel valued and appreciated.” Another wrote, “I thought of comforting moments. Where my partner was caring for me, giving me extra attention and comfort because I was feeling down.”

#### Theme 3: item interpretations

Finally, many participants (74.07%) attempted to infer the meaning of one or more items on the basis of the focal word within the item, usually drawing on its connotations or associations with other concepts. Frequently, participants would then relate the term back to their own relationships. For example, when explaining why they rated the snowdrop item positively, one participant wrote, “it likely means finding joy, love, and beauty in the small, precious moments or gestures I receive from our relationship.” In response to the umbra item, one participant wrote, “To me umbra means natural, genuine, and wholehearted. I love my man and that’s exactly what our relationship is to me.”

### Study 1 discussion

Study 1 illustrates how sentiment override can muddy the interpretation of relationship findings. Study 1a showed that despite being meaningless on its face, Pseudo had satisfactory psychometric properties and was effective at predicting relationship outcomes in the context of a weekly experience study. Qualitative probing (Study 1b) suggested that this likely occurs because participants imbue the Pseudo items with relational meaning, particularly by projecting their positive feelings about the relationship onto the items (i.e., sentiment override; Fincham et al., 1995; Weiss, 1980). Together, these findings suggest that self-report relationship measures can produce seemingly meaningful results (e.g., predicting weekly relationship outcomes) in the absence of novel, substantive meaning, simply by tapping into general relationship sentiments.

## Study 2

In Study 2, we quantitively probed the properties of Pseudo using a dyadic longitudinal design. Does Pseudo indeed function as a measure of current global sentiment? And if so, how does it differ from established relationship-quality measures?

Sentiment override is a relationship-specific rather than domain-general source of method bias. Therefore, we expected Pseudo to be strongly associated with other relationship measures (e.g., satisfaction, commitment, *r*s ~ .40), and weakly associated with nonrelationship variables (e.g., the Big Five; *rs* < .20). Further, we preregistered that Pseudo would be weakly associated between partners (i.e., that it captures a relatively individual phenomenon) and that it would vary over time. These features should set Pseudo apart from established relationship constructs (e.g., satisfaction, commitment), which are intended to capture more stable, dyadic relationship features. Preregistrations, materials, and analysis scripts are publicly available (https://osf.io/sprgj/).

### Method

#### Participants and procedure

Participants were recruited from the United Kingdom via research volunteer panels, online advertisements, and printed flyers. Eligible partners were at least 18 years old; fluent in English; currently in a relationship of at least 3 months’ duration; saw their partner in person at least 5 days per week; and had regular Internet access. Data collection began in September 2022 and finished in February 2023. The study had five phases in total. The current analyses used self-report data from three phases (1, 4, and 5).

The baseline sample consisted of 126 couples (103 man-woman dyads, 10 woman–woman, 3 woman–agender/genderless, 1 woman–nonbinary/genderqueer, 3 man–man, 4 man–nonbinary/genderqueer, 2 unknown) with a mean age of 32 (*SD* = 13.88 years, range = 19–78 years) and a mean relationship length of 8 years (*SD* = 10.89 years, range = 0.33–52.5 years). Participants were primarily heterosexual (*n* = 175, 69.4%), White (*n* = 210, 83.3%), cisgender (*n* = 237, 94.0%), exclusively dating their partner (*n* = 119, 47.2%), and living together (*n* = 196, 77.8%). See Table S4 in the Supplemental Material for detailed participant demographics.

Of the 126 couples who completed the baseline survey, five couples dropped out of the study before Phase 2, seven dropped out by Phase 4, and two broke up by Phase 5. There were also couples for whom only one partner provided data (*n* = 2 at baseline, *n* = 9 at Phase 4, and *n* = 6 at Phase 5). In total, longitudinal analyses included data from 250 individuals at baseline (124 complete dyads), 215 individuals at Phase 4 (103 complete dyads), and 214 individuals at Phase 5 (104 complete dyads), all of which would meet sample-size requirements for modeling a small number of well-defined latent variables (MacCallum et al., 1999), including in the presence of meaningful dependency (Little, 2013).

#### Measures

Pseudo was measured at each wave with the following five items: “I love the snowdrop that I get from my relationship,” “I appreciate the umbra that my relationship brings,” “My partner and I have excellent taro,” “The daffodil in my relationship is close to ideal,” and “I treasure the reseda in my relationship.” The items were measured on a Likert-type scale ranging from 1, *strongly disagree*, to 7, *strongly agree* (Phase 1: α = .88).

##### Relationship measures

The Phase 1 survey included 12 items from the PRQC scale (Fletcher et al., 2000), with three items capturing satisfaction (e.g., “How satisfied are you with your relationship?”, α = .96), three items capturing commitment (e.g., “How dedicated are you to your relationship?”, α = .94), three items capturing trust (e.g., “How much can you count on your partner?”, α = .80), and three items capturing intimacy or closeness (e.g., “How intimate is your relationship?”; α = .86). Perceived partner responsiveness (Crasta et al., 2021) was measured with four items (e.g., “My partner really listens to me”; α = .88). Four items from the Positive and Negative Relationship Quality scale (PN-RQ; Rogge et al., 2017) captured positive relationship quality (e.g., “pleasant,” “strong”; α = .89), and four items captured negative relationship quality (e.g., “miserable,” “lifeless”; α = .75). These items were all measured on a scale ranging from 0, *not at all*, to 6, *completely*.

Six items from the Experiences in Close Relationships scale (ECR-12; Lafontaine et al., 2015) captured *attachment anxiety* (e.g., “I need a lot of reassurance that I am loved by my partner”; α = .85), and six items captured *attachment avoidance* (e.g., “I don’t feel comfortable opening up to romantic partners”; α = .84) on a scale ranging from 1, *strongly disagree*, to 7, *strongly agree. Relationship mindfulness* was measured with 15 ad hoc items (e.g., “When I am upset with my partner, I notice how I am feeling before responding”; Gazder & Stanton, 2023; α = .75) on a scale ranging from 0, *not at all true*, to 6, *completely true.* Finally, satisfaction and commitment were also measured at follow-up (Phases 4 and 5), using the same items reported at baseline.

##### Nonrelationship measures

The Big Five personality traits were measured at Phase 1 with 15 items that begin with the sentence stem “I am someone who . . .” (BFI-2-XS; Soto & John, 2017), with three items capturing agreeableness (e.g., “is compassionate, has a soft heart”; α = .56), three items capturing extraversion (e.g., “is full of energy”; α = .59), three items capturing openness (e.g., “is fascinated by art, music, or literature”; α = .69), three items capturing neuroticism (e.g., “worries a lot”; α = .73) and three items capturing conscientiousness (e.g., “is reliable, can always be counted on”; α = .67). The items were measured on a scale ranging from 1, *strongly disagree*, to 7, *strongly agree.* General mindfulness was measured with 15 items (e.g., “I’m good at finding words to describe my feelings”; Baer et al., 2008; α = .77) rated on a scale ranging from 0, *not at all true*, to 6, *completely true*. Anxiety symptoms (Spitzer et al., 2006; α = .91) and depressive symptoms (Kroenke et al., 2001; α = .87) were measured with seven and nine items, respectively, capturing the occurrence of problems over the past 2 weeks (e.g., “Feeling nervous, anxious or on edge”; “Little interest or pleasure in doing things”), on a scale ranging from 0, *not at all*, to 3, *nearly every day*. Satisfaction with life (Diener et al., 1985) was captured with five items (e.g., “The conditions of my life are excellent”; α = .85) on a scale ranging from 1, *strongly disagree*, to 7, *strongly agree*. Finally, meaning in life (Costin & Vignoles, 2020) was captured with four items (e.g., “My life as a whole has meaning”; α = .74) on a scale ranging from 1, *strongly disagree*, to 7, *strongly agree*.

### Results

#### Analytic strategy

Means, standard deviations, and correlations are shown on Table 5 (relationship variables) and Table 6 (nonrelationship variables). These bivariate analyses initially suggest that Pseudo shares moderate to strong associations with relationship measures (consistent with Study 1) and weak or null associations with nonrelationship measures.

**Table 5.** Correlations Between Relationship Measures in Study 2

Variable	*M*	*SD*	1	2	3	4	5	6	7	8	9	10
1. Pseudo	5.21	1.00										
2. Satisfaction	4.95	1.04	.42**									
3. Commitment	5.37	0.83	.35**	.72**								
4. Trust	5.25	0.83	.28**	.60**	.57**							
5. Closeness	4.85	0.96	.40**	.76**	.63**	.48**						
6. Perceived partner responsiveness	4.66	1.10	.35**	.72**	.52**	.53**	.63**					
7. Positive relationship quality	4.97	0.89	.39**	.82**	.64**	.50**	.73**	.71**				
8. Negative relationship quality	0.49	0.60	−.28**	−.64**	−.44**	−.43**	−.56**	−.54**	−.61**			
9. Attachment anxiety	3.94	1.41	.11	−.08	−.03	−.17**	.05	−.08	.01	.16*		
10. Attachment avoidance	2.29	1.01	−.30**	−.43**	−.39**	−.35**	−.45**	−.42**	−.43**	.32**	−.04	
11. Relationship mindfulness	3.53	0.66	.26**	.42**	.37**	.19**	.45**	.36**	.39**	−.27**	−.05	−.33**

*p < .05. **p < .01.

**Table 6.** Correlations between nonrelationship measures in Study 2

Variable	*M*	*SD*	1	2	3	4	5	6	7	8	9	10
1. Pseudo	5.21	1.00										
2. Extraversion	4.31	1.16	.02									
3. Agreeableness	5.20	1.00	.17**	−.04								
4. Conscientiousness	4.63	1.31	.02	.18**	.14*							
5. Neuroticism	4.16	1.36	.15*	−.23**	−.11	−.40**						
6. Openness	5.13	1.16	.02	.26**	−.03	−.13*	.12					
7. General mindfulness	3.28	0.75	−.04	.14*	.13*	.46**	−.50**	.15*				
8. Anxiety symptoms	5.99	5.17	.08	−.14*	−.10	−.36**	.66**	.12	−.47**			
9. Depressive symptoms	5.81	5.28	.01	−.20**	−.07	−.40**	.57**	.09	−.44**	.74**		
10. Satisfaction with life	4.89	1.21	.16*	.25**	.14*	.36**	−.35**	.04	.31**	−.47**	−.56**	
11. Meaning in life	5.30	1.03	.12	.34**	.21**	.34**	−.41**	.12	.44**	−.37**	−.52**	.57**

*p < .05. **p < .01.

We examined these associations further with a series of dyadic SEM analyses with latent variables (Sakaluk et al., 2025). The data were structured in dyadic format (one row per couple), and the dyads were treated as distinguishable, with actor and partner roles assigned randomly for each couple. All models were fitted using the *lavaan* package in *R* (Rosseel, 2012). We preregistered dozens of confirmatory models in two separate phases (baseline and longitudinal). We report only the key models here; the remainder are available in the Supplemental Material. All models were preregistered unless otherwise noted.

#### Associations between Pseudo and other relationship measures

**Fig. 1.**
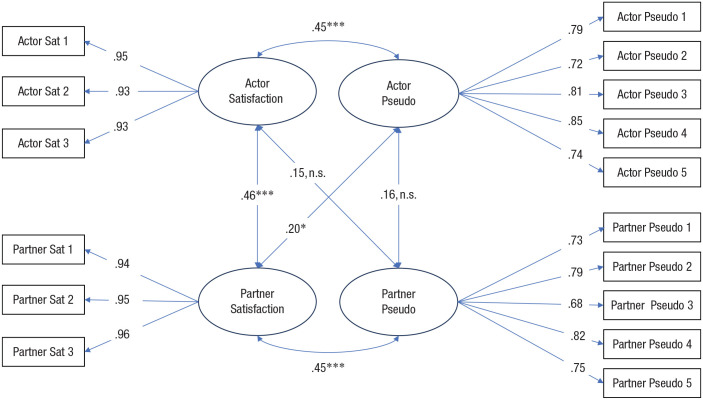
Dyadic associations between latent satisfaction and latent Pseudo. Sat = satisfaction. Residual variances and covariances (between the same item across partners) are not depicted for figure clarity. *p < .05. ***p < .001.

We probed Pseudo’s association with each of the 10 available relationship-specific measures (e.g., satisfaction, commitment, attachment style). We predicted that Pseudo would have moderate to strong latent associations with each of the relationship factors (*r* = .40), suggesting that Pseudo is tapping into relationship-specific evaluations. Results for all relationship variables can be seen in Table 7, with the satisfaction results depicted as an example in Figure 1. Indeed, latent Pseudo was moderately correlated with most latent relationship factors, with a median absolute *r* of .378 (range = .018–.461). For nine out of 10 relationship measures tested, constraining the correlations to .40 (or −.40, for negatively valanced constructs) did not result in significantly worse fit, suggesting that these latent associations were of the predicted size. The only exception was attachment anxiety, which suggests that attachment anxiety has little conceptual overlap with Pseudo.

**Table 7.** How Strongly Is Pseudo Associated With Relationship Constructs?

Latent construct	Correlations with latent Pseudo	Comparing constrained vs. unconstrained models	
		Δχ^2^ (*df diff* = 2)	*p*
Satisfaction	.452 (*a*), .450 (*p*)	.875	.646
Commitment	.325 (*a*), .375 (*p*)	.910	.635
Trust	.380 (*a*), .229 (*p*)	3.623	.163
Closeness	.436 (*a*), .448 (*p*)	.504	.777
Responsiveness	.337 (*a*), .432 (*p*)	.675	.714
Positive relationship evaluations	.407 (*a*), .461 (*p*)	.455	.797
Negative relationship evaluations	−.346 (*a*), −.286 (*p*)	1.654	.437
Attachment anxiety	.018 (*a*), .112 (*p*)	19.153	< .001
Attachment avoidance	−.242 (*a*), −.418 (*p*)	2.731	.255
Relationship mindfulness	.256 (*a*), .419 (*p*)	1.698	.428

Note: In the constrained models, correlations between latent factors were constrained to .4. Correlations were examined separately for the latent actor variables (e.g., actor satisfaction and actor Pseudo), denoted with *a*, and the latent partner variables (e.g., partner satisfaction and partner Pseudo), denoted with *p*.

#### Associations between Pseudo and nonrelationship measures

**Fig. 2.**
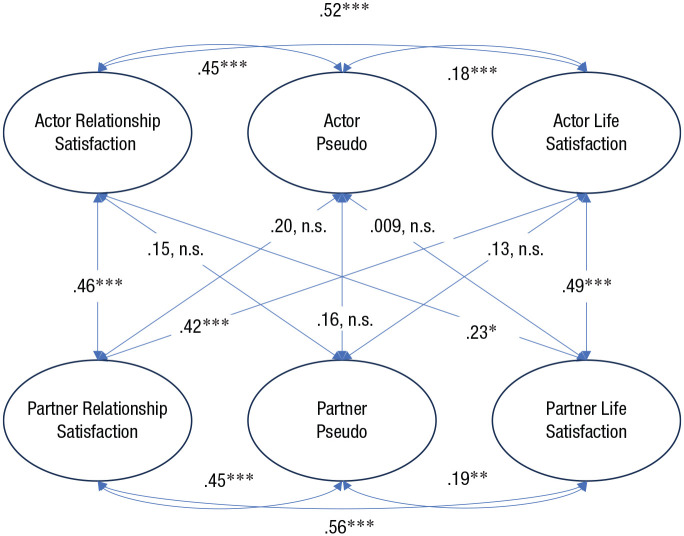
Dyadic associations between latent relationship satisfaction, satisfaction with life, and Pseudo. For clarity, the measurement model is not shown. *p < .05. **p < .01. ***p < .001.

We next examined Pseudo’s overlap with measures that are not about close relationships. We predicted that Pseudo would be significantly more strongly associated with relationship measures compared with nonrelationship measures, which suggests that Pseudo is tapping into a relationship-specific bias. We tested this with a series of models pairing one relational construct with one nonrelational construct and examining their respective associations with Pseudo. Results can be seen in Table 8, with an example model depicted in Figure 2. Hypotheses were generally supported, as nearly all correlations between Pseudo and relational measures were descriptively higher than the nonrelational measures. Constraining paths for the relational versus nonrelational factors to equal resulted in significantly worse fit in six out of nine models tested. These results suggest that Pseudo was primarily tapping into relational biases, rather than more general (nonrelational) biases.

**Table 8.** How Strongly Is Pseudo Associated With Relational Versus Nonrelational Constructs?

Relational variables		Nonrelational variables		Comparing constrained vs. unconstrained models	
Latent construct	Correlations with latent Pseudo	Latent construct	Correlations with latent Pseudo	Δχ2 (*df diff* = 2)	*p*
Satisfaction	.448 (*a*), .451 (*p*)	Satisfaction with life	.176 (*a*), .193 (*p*)	18.181	< .001
Closeness	.434 (*a*), .438 (*p*)	Meaning in life	.129 (*a*), .047 (*p*)	15.602	< .001
Positive relationship evaluations	.403 (*a*), .462 (*p*)	Life satisfaction	.170 (*a*), .199 (*p*)	10.772	.005
Negative relationship evaluations	−.343 (*a*), −.308 (*p*)	Depression	−.031 (*a*), .108 (*p*)	15.34	< .001
Attachment anxiety	.130 (*a*), .187 (*p*)	Generalized anxiety	.050 (*a*), .184 (*p*)	.537	.765
Attachment anxiety	.021 (*a*), .111 (*p*)	Neuroticism	.069 (*a*), .299 (*p*)	3.577	.167
Attachment avoidance	.242 (*a*), .420 (*p*)	Agreeableness	.365 (*a*), .098 (*p*)	3.571	.168
Relationship mindfulness	.249 (*a*), .418 (*p*)	General mindfulness	−.072 (*a*), .141 (*p*)	6.12	.047
Satisfaction	.451 (*a*), .451 (*p*)	Meaning in life	.127 (*a*), .047 (*p*)	21.196	< .001

Note: In the constrained models, correlations between the Pseudo factor and the relational versus nonrelational factors were constrained to be equal. All models were preregistered except for the model comparing satisfaction to meaning in life.

### Interpartner agreement on Pseudo (exploratory)

**Fig. 3.**
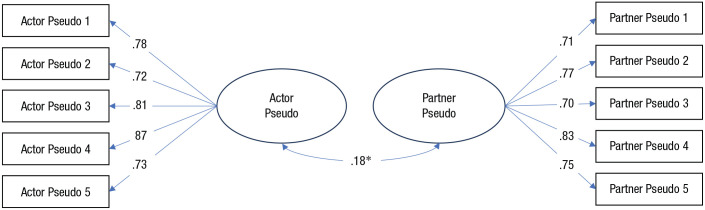
Interpartner agreement for Pseudo. For clarity, residual variances and covariances (between the same item across partners) are not depicted. *p < .05.

We conducted several exploratory models examining interpartner agreement on appraisals of relationship-specific constructs. For each target measure, we loaded the relevant items onto a latent factor for each partner and examined the association between the latent factors. We found that partners had moderate to high agreement on satisfaction (*r* = .454), commitment, (*r* = .313), closeness (*r* = .275), perceived partner responsiveness (*r* = .331), positive relationship quality (*r* = .372), and negative relationship quality (*r* = .320) within their relationships (median *r* = .326, *SD* = .06). In contrast, partners had relatively low agreement on Pseudo (*r* = .179; see Fig. 3).

### Stability of Pseudo over time

How stable is Pseudo over time? We tested a preregistered latent model in which the Pseudo items from each of the three time points were loaded onto a single factor; we then compared it to a model in which items were loaded onto a separate factor for each time point. The single factor model fit the Pseudo items poorly, χ^2^(359) = 972.203, *p* < .001, CFI = .764, RMSEA = .116, SRMR = .087, whereas a model with a separate latent Pseudo factor for each time point fit significantly better, Δχ^2^ = 162.56, *df diff* = 14, *p* < .001. These results suggest that Pseudo is not particularly traitlike or stable over time. However, it is important to note that we found the same results when examining satisfaction (confirmatory) as well as commitment (exploratory). In each case, a one-factor solution fit the data poorly, whereas a three-factor solution fit significantly better. We also examined the latent stabilities for each variable. Pseudo’s stabilities between time points (*M* = .726, *SD* = .096, range = .632–.841) were similar to those of commitment (*M* = .767, *SD* = .070, range = .683–.868) and satisfaction (*M* = .775, *SD* = .031, range = .74–.821). These results suggest that Pseudo’s stability is similar to that of more established relationship constructs.

### Study 2 discussion

Study 2 results suggest that Pseudo primarily captures sentiment override. Pseudo was moderately associated with relationship constructs but was weakly associated with nonrelationship constructs, suggesting that Pseudo primarily taps into relationship-specific appraisals. However, whereas romantic partners tended to agree on established relationship measures—indicative of interdependent, dyadic phenomena (Rusbult & Van Lange, 2008)—agreement on Pseudo was relatively absent, indicative of an intraindividual phenomenon (e.g., response biases). Finally, Pseudo was not particularly stable over time, consistent with the idea that Pseudo is capturing people’s fleeting feelings toward their partner (although other relationship measures similarly lacked stability over time).

## General Discussion

The study of close relationships is one of the largest thematic undertakings across areas of psychological inquiry and heavily relies upon the usage—and therefore validity—of self-report measures (Williamson et al., 2022). The current research highlights how relationship research may be vulnerable to the confounding influence of method biases. We constructed a scale consisting of irrelevant relationship evaluation items (Pseudo). In Study 1a, Pseudo displayed desirable psychometric properties and significantly predicted other relationship measures over time. In Study 1b, qualitative probing suggested that participants tended to imbue the Pseudo items with relational meaning. In Study 2, Pseudo behaved similarly to established relationship-quality measures like satisfaction, with the exception that romantic partners did not agree on Pseudo. Overall, results suggest there are few differences between Pseudo and any number of ostensibly novel, useful relationship measures, despite Pseudo lacking substantive item content.

Our findings are most consistent with the idea that Pseudo captures global relationship sentiments. In line with the processes of sentiment override (Weiss, 1980), and conversational implicature (the idea that people infer meaning using conversational norms; Schwarz, 1994), people likely attempt to make sense of Pseudo by projecting their relationship feelings onto those items. This interpretation is supported by qualitative responses from participants (e.g., “I wasn’t sure what umbra meant. But I appreciate everything that comes with my relationship, so I clicked ‘agree.’”). This interpretation of the results is concerning, because although global sentiments are valid, they are not novel. The current research certainly did not reveal an exciting new way to improve people’s relationships. Yet Pseudo was able to produce the appearance of evidence for incremental validity as though it represented a promising new construct. These findings suggest that relationship measures can appear to capture something novel simply by tapping into global relationship evaluations. This is particularly concerning given recent research suggesting that most relationship measures tap into global relationship evaluations, at least partially if not primarily (Kim et al., 2025).

Alternatively, Pseudo may capture domain-general method biases (Podsakoff et al., 2003), such as a halo effect (e.g., Nisbett & Wilson, 1977), self-enhancement (e.g., Sedikides & Gregg, 2008), or acquiescence (e.g., Van Herk et al., 2004). It seems unlikely that Pseudo is primarily domain-general given its weak associations with nonrelationship measures (Study 2). Nevertheless, if we entertain this interpretation, it suggests that the methods employed in the current studies do not sufficiently distinguish between conceptual signal and largely domain-irrelevant methodological artifacts.

### Recommendations for the future study of interpersonal relationships

Overall, the current findings suggest that method biases pose a threat to the validity of interpersonal self-report measures. This presents serious challenges for the testing of interpersonal-relationship theories, which assume that relationship measures are distinctive, accurate representations of their intended constructs. We offer several strategies for moving forward in light of these concerns.

#### Curation of measures

Given Pseudo’s prominent confounding effects in our studies and the compelling demonstrations of measurement-related problems in psychology (e.g., Elson et al., 2023; Flake & Fried, 2020; Hussey & Hughes, 2020), we are calling for a focused effort to scrutinize self-report measures of close relationships. The sheer number of interpersonal measures has grown unreasonably large, leading to conceptual confusion (e.g., Rosenbusch et al., 2020), and little measurement consistency (CORE Lab, 2025; Elson et al., 2023). We recommend a culling process in which the field’s measures and constructs are carefully interrogated, ideally with large-scale multilab studies (e.g., “ManyLabs”; Klein et al., 2014). The preferred measure of a given construct should be selected and widely agreed upon, and alternative measures should be retired (e.g., see APA’s guidelines for measuring depression: https://www.apa.org/depression-guideline/assessment). Further, the bar for introducing new measures to the literature should be appropriately increased.

#### Greater adoption of qualitative approaches

Measures should be carefully reexamined for content validity (e.g., Clifton, 2020), particularly with often-overlooked qualitative methods that probe participants’ interpretation (Wolf, 2023), such as cognitive probing (Behr et al., 2017). Such methods are invaluable for revealing how participants actually arrive at their responses.

#### Greater adoption of SEM approaches

SEM is an important tool for combating measurement threats, such as method biases (e.g., Sakaluk et al., 2025; Wang & Eastwick, 2020). The field would therefore benefit from a more careful interrogation of measurement models with the use of SEM, particularly in dyadic contexts (e.g., Little, 2013; Sakaluk et al., 2021, 2025). Within an SEM framework, researchers could explicitly test for the presence of method factors like sentiment override (e.g., Podsakoff et al., 2003, 2024), using, for example, data from multiple partners and objective raters (a multitrait-multimethod approach; e.g., Eid et al., 2008). It is even possible that Pseudo could function as a measured cause variable, allowing global relationship evaluations to be incorporated into latent models as a method factor (the directly measured latent-variable technique; see Simmering et al., 2015, for a review).

#### New resources for researchers and reviewers

We can encourage the adoption of more rigorous measurement testing by developing user-friendly resources. For example, open-source functionality could be created to help researchers test for method factors. Accessible-method tutorial papers could further explain the importance of method biases and lay out patterns of effects that would confirm their presence (or not). Simultaneously, checklists of measurement- and modeling-related issues to look for could be provided to reviewers, particularly for appraising more psychometric-oriented papers (e.g., those presenting a new scale).

### Conclusions

The current findings demonstrate how method biases can threaten the validity of findings from the psychological study of interpersonal relationships when using self-report measures. Meaningfully addressing this problem will require a renewed focus on basic measurement and validation research, as well as the adoption of modeling approaches that can help disentangle theoretical signal from methodological confounds.

## Supplemental Material

sj-docx-1-pss-10.1177_09567976251370262 – Supplemental material for Pseudo Effects: How Method Biases Can Produce Spurious Findings About Close Relationships Supplemental material, sj-docx-1-pss-10.1177_09567976251370262 for Pseudo Effects: How Method Biases Can Produce Spurious Findings About Close Relationships by Samantha Joel, John K. Sakaluk, James J. Kim, Devinder Khera, Helena Yuchen Qin and Sarah C. E. Stanton in Psychological Science

## Transparency

*Action Editor:* Yoel Inbar

*Editor:* Simine Vazire

*Author Contributions*

**Samantha Joel:** Conceptualization, Formal analysis, Funding acquisition, Methodology, Project administration, Resources, Writing – original draft, Writing – review & editing.

**John K. Sakaluk:** Conceptualization, Formal analysis, Methodology, Writing – original draft, Writing – review & editing.

**James J. Kim:** Conceptualization, Methodology, Writing – review & editing.

**Devinder Khera:** Data curation, Investigation, Project administration, Writing – review & editing.

**Helena Yuchen Qin:** Data curation, Formal analysis, Methodology.

**Sarah C. E. Stanton:** Data curation, Funding acquisition, Investigation, Project administration, Resources, Writing – review & editing.

**Artificial Intelligence:** No artificial-intelligence-assisted technologies were used in this research or the creation of this article.

**Ethics:** Study 1 received approval from the Western University Non-Medical Research Ethics Board (Project ID No. 119231), and Study 2 received approval from the University of Edinburgh PPLS Research Ethics Committee (Ref. No. 422-2122/1). Computational reproducibility: The computational reproducibility of the results in the main article (but not the Supplemental Material) has been independently confirmed by the journal’s STAR team. Open Science Framework (OSF): To ensure long-term preservation, we registered all OSF files at https://osf.io/8zh9d.

**Open Practices:** Pilot study disclosures. Preregistration: The pilot study was not preregistered. Materials: All materials are publicly available (https://osf.io/vjnrp/). Data: All primary data are publicly available (https://osf.io/vjnrp/). Analysis scripts: All analysis scripts are publicly available (https://osf.io/vjnrp/). Study 1a disclosures. Preregistration: Study 1a was not preregistered. Materials: All materials are publicly available (https://osf.io/2tqke/). Data: All primary data are publicly available (https://osf.io/2tqke/). Analysis scripts: All analysis scripts are publicly available (https://osf.io/2tqke/). Study 1b disclosures. Preregistration: Study 1b was not preregistered. Materials: All materials are publicly available (https://osf.io/knca8/). Data: All primary data are publicly available (https://osf.io/knca8/). Analysis scripts: All analysis scripts are publicly available (https://osf.io/knca8/). Study 2 disclosures. Preregistration: The hypotheses and analysis plan regarding the baseline data were preregistered on March 27, 2023: https://osf.io/jpc6z. The hypotheses and analysis plan regarding the longitudinal data were preregistered on September 1, 2023: https://osf.io/mc52r. In each case, the preregistration was submitted after data collection was complete, but before the first and second authors had access to the data. Data were collected between October 2022 and February 2023. There were minor deviations from the preregistered plan, which are reported in the supplemental file. Materials: All materials are publicly available (https://osf.io/sprgj/). Data: Data are not publicly available because of confidentiality concerns. Data are available upon request from S. C. E. Stanton (Sarah.Stanton@ed.ac.uk). Analysis scripts: All analysis scripts are available (https://osf.io/sprgj/).

## Notes

1. Note that this data set is also reported in Joel et al. (2023). No previously published analyses have included the Pseudo items.
