# Supplementary material for: Pseudo Effects: How Method Biases Can Produce Spurious Findings About Close Relationships
Source: Psychol Sci. 2025 Sep 10;36(10):780–95. doi: 10.1177/09567976251370262 (PMC13428953; doi:10.1177/09567976251370262)
Supplement: Supplementary file 1 [file sj-docx-1-pss-10.1177_09567976251370262.docx]

**Pilot Sample Supplemental Materials**

The purpose of the pilot study (Study 0 on OSF) was to develop and subject the Pseudo scale to initial validation testing. We constructed 10 items that superficially appear to be evaluative of a relationship, but the content of which does not tap into any real or meaningful aspect of the relationship (e.g., “I enjoy the dolomite that I share with my partner”). We did this by drawing on common relationship-evaluative items from established relationship measures (e.g., “My partner and I have excellent _____”, “I treasure the _____ in my relationship”). We then replaced each target noun, which would typically be a relationship feature (e.g., love, communication, passion), with a relationship-irrelevant term, such as the name of a plant or mineral (e.g., daffodil, snowdrop, umbra, terra). We administered these items to an online sample of people in romantic relationships and examined their psychometric properties. This pilot study was not preregistered. Data, code, and materials are available at https://osf.io/vjnrp/.

**Method**

**Participants**

We posted the study on Western University’s Mass Email Recruitment listserv and on Reddit in Spring 2022, in exchange for an entry into a draw for one of four $25.00 CAD/USD Amazon Gift Cards. We ceased recruitment after June 5th, 2022, when 455 participants had begun the study and 393 participants had completed the study. We excluded 64 participants for failing one of two attention checks and an additional 37 participants who withdrew during debriefing. The final sample was 331 participants (118 men, 186 women, 27 non-binary) with a mean age of 25 years (SD = 7.63, range = 18-63). The participant sample was primarily heterosexual (n = 184; 55.6%), white (n = 238; 71.9%), cisgender (n = 303; 91.5%), and were in non-married committed relationships (n = 217; 65.6%). See Supplemental Table 1 for detailed participant demographics.

**Procedure and Measures**

**Pseudo items.** Participants were shown the following prompt: “These next questions may seem a bit strange. It’s okay if you’re not sure what they mean. Please answer them anyway to the best of your ability”. They were then presented with 10 pseudo-evaluative relationship items (e.g., “My partner and I have excellent taro”) on a scale from 1 = *strongly disagree* to 7 = *strongly agree.* These items included five terms selected for being widely recognizable (e.g., Saturn, daffodil, midnight) and five that we expected to be less recognizable (e.g., fuchsine, reseda, umbra). Participants also completed demographic questions and several other measures intended for other research purposes.

**Pilot Sample Results**

We first examined the factor structure of the 10 Pseudo items following recommended procedures for EFA (Fabrigar & Wegener, 2011, Sakaluk & Short, 2017). A parallel analysis (Horn, 1965) using the Psych package (Revelle, 2023) in R (R Core Team, 2023) suggested the presence of one factor. An exploratory factor analysis constrained to one factor accounted for 58% of the variance, and all 10 items loaded highly onto the single factor, all loadings > .70, suggesting that this one-factor solution was well-suited to the data (e.g., Tabachnick & Fidell, 2019). Metrics of model fit further suggested that this was a reasonably acceptable model, given our moderate sample size, RMSEA = .10, TLI = .93.

The final scale is displayed on Table 1 of the main text. Most items were distributed relatively normally (*skews* = -.33 to -.52), with means only slightly above the midpoint of the scale. All items had responses spanning the entire scale (all *range*s = 1 to 7). Given their high factor loadings and acceptable psychometric properties, all 10 items were retained in the final scale (α = .93).

**Supplemental Table 1**

*Detailed pilot study participant demographics*

|  | ***n* (%)** |
| --- | --- |
| **Gender/Sex** |  |
| Cisgender | 303 (91.5) |
| Transgender | 2 (0.6) |
| Intersex | 26 (7.9) |
| **Gender** |  |
| Man | 118 (35.6) |
| Woman | 186 (56.2) |
| Non-binary | 27 (8.2) |
| **Sexual Orientation** |  |
| Straight | 184 (55.6) |
| Gay | 12 (3.6) |
| Lesbian | 20 (6.0) |
| Bisexual | 83 (25.1) |
| Pansexual | 23 (7.0) |
| Asexual | 9 (2.7) |
| **Relationship Status** |  |
| Casually dating | 49 (14.8) |
| Non-married committed relationship | 217 (65.6) |
| Married | 65 (19.6) |
| **Ethnicity/Race** |  |
| African/Black | 5 (1.5) |
| White | 238 (71.9) |
| South Asian | 13 (3.9) |
| Asian/East Asian | 29 (8.8) |
| Indigenous | 2 (0.6) |
| Hispanic/Latinx | 7 (2.1) |
| Middle Eastern | 4 (1.2) |
| Multiethnic | 28 (8.5) |
| Prefer not to say | 5 (1.5) |
| **Education** |  |
| Some high school | 4 (1.2) |
| High school diploma | 39 (11.8) |
| Some college/university | 137 (41.4) |
| Completed undergraduate degree | 111 (33.5) |
| Vocational degree/certificate | 6 (1.8) |
| Post-graduate studies | 34 (10.3) |

**Validation Evidence Review Materials**

Qualitative validation methods are uncommon within relationship science. At the suggestion of a reviewer, we sought to illustrate this point by coding 44 common relationship measures for qualitative validity evidence. We used the relationship-specific measures selected by Kim et al. (2024), which were chosen to represent the most common predictors of satisfaction in Joel et al. (2020). These measures are all widely cited and used within the field.

We based our coding scheme on the Standards of Educational and Psychological Testing (2014 edition) as suggested by the reviewer. The Validity chapter of this text (pp. 11-26) describes four potential sources of validity evidence for a new measure or test. Two of those sources of evidence are the quantitative sources of evidence that the current paper is focused on (e.g., factor analyses, convergent validity, predicting relevant outcomes). The other two sources are qualitative sources of evidence regarding the test content (what is the content of the items?), and response processes (how do people interpret and respond to the items?).

For each of the 44 relationship measures, the first author read the paper in which the scale was originally published, and documented any validity evidence regarding 1) item content and 2) response processes. Results are presented in an additional supplemental Excel file shared on OSF within the Pilot Data component (“Coding of Common Relationship Scales.xlsx”; <https://osf.io/vjnrp/>). We found that 16 measures (36%) presented what we consider to be reasonable evidence of content validity, such as having selected the items based on a panel of experts (e.g., marital therapists), interviews with participants (e.g., couples), or from a pool of existing measures within the literature. The remaining 28 measures (64%) did not present evidence of content validity. Most commonly, the items were simply generated by the researchers, and the content of the scales was not probed any further.

Response evidence, where participants were asked to give feedback on the items, was presented for only three of the 44 measures (7%). In particular, it was exceedingly rare for relationship scientists to directly ask participants what they think a measure means (e.g., using cognitive interviewing). This kind of qualitative data was available for only one of the 44 measures we examined (the Inclusion of Other in Self Scale; Aron et al., 1992). Overall, these results provide some initial evidence that relationship science has generally overlooked the importance of qualitative evidence in the scale development process.

**Study 1B Supplemental Materials**

The purpose of Study 1b was to qualitatively probe how participants were interpreting the Pseudo items. To what extent did participants attempt to infer meaning from the seemingly meaningless items? Participants were asked to rate three Pseudo items in a randomized order. Following each item, participants were asked to explain their reasoning via open-ended text boxes. Specifically, they were asked why they chose the rating they chose, what they were thinking about while completing the item, and what they thought the item meant. We coded then these open-ended responses for themes. Due to limited page space, we describe this study only briefly in the main text, with full methodological details available here.

**Method**

**Participants**

Participants were recruited on Western University’s Mass Email Recruitment listserv in June 2024, in exchange for an entry into a draw for one of four $70.00 CAD or $50.00 USD Amazon gift cards. A total of 101 participants responded to at least one survey question. 18 participants were excluded for providing no substantive responses in all open-ended questions, and two additional participants were excluded due to being single. The final sample was 81 participants (10 men, 37 women, 1 non-binary, 33 who did not specify their gender), with a mean age of 24.77 years (*SD* = 6.11, range = 18-46). The participants were primarily heterosexual (n = 31; 38.27%), white (n = 27; 33.33%), and were in non-married committed relationships (n = 31; 38.27%). See Supplemental Table 2 for detailed participant demographics.

**Supplemental Table 2**

*Detailed Pilot Study B Participant Demographics*

|  | ***n* (%)** |
| --- | --- |
| **Gender** |  |
| Man | 10 (12.35) |
| Woman | 37 (45.68) |
| Non-binary | 1 (1.23) |
| No entry | 33 (40.74) |
| **Sexual Orientation** |  |
| Straight | 37 (45.68) |
| Gay | 0 (0) |
| Lesbian | 1(1.23) |
| Bisexual | 5 (6.17) |
| Pansexual | 2 (2.47) |
| Asexual | 1 (1.23) |
| Prefer to self-describe | 1 (1.23) |
| No entry | 34 (41.98) |
| **Relationship Status** |  |
| Casually dating | 7 (8.64) |
| Non-married committed relationship | 31 (38.27) |
| Married/Civil Union | 9 (11.11) |
| No entry | 34 (41.98) |
| **Ethnicity/Race** |  |
| African/Black | 2 (2.47) |
| White | 27 (33.33) |
| South Asian | 6 (7.41) |
| Asian/East Asian | 8 (9.88) |
| Indigenous | 0 (0) |
| Hispanic/Latinx | 2 (2.47) |
| Middle Eastern | 4 (4.94) |
| Pacific Islander | 0 (0) |
| Multiethnic | 1 (1.23) |
| Prefer to self-describe | 1 (1.23) |
| Prefer not to say | 2 (2.47) |
| No entry | 34 (41.98) |
| **Education** |  |
| Some high school | 0 (0) |
| High school diploma | 0 (0) |
| Some college/university | 18 (22.22) |
| Completed undergraduate degree | 9 (11.11) |
| Vocational degree/certificate | 0 (0) |
| Post-graduate studies | 20 (24.69) |
| No entry | 34 (41.98) |

**Procedure and Measures**

As part of a broader study, participants were randomly assigned to complete one of the three sets of questions, with items focusing either on satisfaction, relationship behaviors, or Pseudo. As only the Pseudo items are relevant to the present paper, we have reported only on the subset of participants assigned to those items (*n* =81). The full questionnaire can be found on OSF (<https://osf.io/knca8/>).

**Pseudo Items*.*** Participants were shown the following prompt: “These next questions may seem a bit strange. It’s okay if you’re not sure what they mean. Please answer them anyway to the best of your ability”. They were then asked to rate each of three Pseudo items: “I love the snowdrop that I get from my relationship”, “I appreciate the umbra that my relationship brings”, and “I treasure the reseda in my relationship”. These three Pseudo items were chosen because they had the highest loadings in previous samples. The order of the Pseudo items was randomized.

**Open-Ended Reasoning.** After each Pseudo item, participants were shown the following prompt:

We are curious about how people answer different survey questions. For the next few questions, we will ask you to share how you came to a specific choice or explain your understanding of the question. Please note that there are no right or wrong answers to these questions. Understanding your reasoning will be useful for us, regardless of what your thought process was.

Participants were then presented with three open-ended questions: a category-selection probe, a specific probe, and a comprehension probe (Behr et al., 2017). The ***category-selection probe*** asked participants to explain why they chose their selected value rather than an adjacent value:

The statement you rated was: [Pseudo item]. Your answer was [response] on a scale from 1 (Strongly disagree) to 7 (Strongly agree). Why did you choose [response], and not the value next to it? For example, if you have chosen a “6”, why didn’t you choose a “5” or a “7”?

Next, the ***specific probe*** asked participants to explain their reasoning in more detail: “The statement you rated was [Pseudo item]. What experiences were you thinking of when you rated this statement?”

Finally, the ***comprehension probe*** asked participants to describe their understanding of the question: “The statement you rated was [Pseudo item]. In your own words, what do you think this statement means?”

**Study 1b Results**

Supplemental Table 3 presents the distribution of response patterns across the three Pseudo items. As is typical for relationship items, the distribution is negatively skewed, with more responses at the higher end of the scale. Further, a non-trivial number of participants chose to leave the Pseudo items blank.

**Supplemental Table 3**

*Response Pattern in Percent (and Absolute Numbers)*

|  | Snowdrop | Umbra | Reseda |
| --- | --- | --- | --- |
| 1 Strongly Disagree | 1.23% (1) | 0% (0) | 0% (0) |
| 2 | 2.47% (2) | 2.47% (2) | 1.23% (1) |
| 3 | 3.70% (3) | 3.70% (3) | 1.23% (1) |
| 4 | 35.80% (29) | 25.93% (21) | 49.38% (40) |
| 5 | 16.05% (13) | 13.58% (11) | 12.35% (10) |
| 6 | 17.28% (14) | 13.58% (11) | 9.88% (8) |
| 7 Strongly Agree | 4.94% (4) | 8.64% (7) | 4.94% (4) |
| No response | 18.52% (15) | 32.10% (26) | 20.99% (17) |

The first and fifth authors first read the open-ended responses for general themes, from which they identified three primary response patterns. First, many participants noted that the items were meaningless, confusing, or otherwise difficult to interpret (e.g., “I have no clue what an umbra is. I looked it up and the definition was not clear.”). Second, many participants drew on their relationships to arrive at a rating for the items (e.g., “I chose this because snowdrop seems like a positive and beautiful thing and I value positivity in my relationship”). Finally, some participants attempted to infer the meaning of the focal words within the items (e.g., “I think probably reseda means trust or honesty”).

We generated a detailed coding scheme based on these themes. Responses were coded along two dimensions: relationship evaluation, and item interpretation. For relationship evaluation, responses were coded based on whether participants drew from their own relationship to arrive at a rating, and if so, whether they mentioned general relationship sentiments and/or specific relationship qualities. For item interpretation, responses were coded based on whether participants explicitly mentioned the confusing nature of the item, as well as whether they attempted to imbue the items with general and/or specific meaning. The first and fifth authors independently coded the open-ended responses according to this scheme, and the codes were finalized by the fifth author. Inter-rater reliability between coders was assessed using unweighted Cohen’s Kappa, with average values for each code ranging from 0.81 to 0.95. Results are presented in Supplemental Table 4 along with kappa values. Drawing on these results, we elaborate on the three general themes below.

**Supplemental Table 4**

*Coding and Reliability of Open-Ended Responses in Pilot Sample B*

|  | Relationship Evaluation | | | Item Interpretation | | | No Substantive  Response |
| --- | --- | --- | --- | --- | --- | --- | --- |
|  | General | Specific | Absent | General | Specific | Meaningless/ Confused |  |
| Snowdrop | 15 (κ = .91) | 25 (κ = .88) | 29 (κ =.92) | 25 (κ = .91) | 31 (κ = .87) | 41 (κ = .95) | 18 (κ = .96) |
| Umbra | 12 (κ = .79) | 19 (κ = .86) | 25 (κ =.88) | 12 (κ = .76) | 29 (κ = .81) | 27 (κ = .91) | 28 (κ = .95) |
| Reseda | 7 (κ = .86) | 12 (κ = .79) | 40 (κ =.88) | 15 (κ = .76) | 25 (κ = .76) | 48 (κ = .95) | 23 (κ = .97) |

*Note****:*** Supplemental Table 4 presents the number of responses assigned to each code, followed by the Cohen’s Kappa. Responses could be assigned multiple codes.

***Theme 1: Confusion*.** Many participants expressed that they did not know what the Pseudo items meant, or that the items were meaningless. Across the sample, 90.12% of the participants expressed uncertainty or confusion in response to at least one of the three Pseudo items, and 17.28% of them expressed uncertainty or confusion in response to all three items. Some participants who expressed confusion nevertheless attempted to interpret the item, while also noting their lack of confidence in their interpretation. For example, when asked to interpret the *reseda* item, one participant wrote, “I can’t fully grasp what this statement means, I believed it was a form of secret resentment towards your partner, but I am not so sure anymore.” Further, in 67.24% of cases where participants expressed confusion about the meaning of an item, they also chose a neutral response for that item (4 “Neither Agree Nor Disagree”). For example, when asked to explain their ratings, one participant wrote, “I chose 4 ‘neither agree nor disagree’ because I don't know what the word reseda means.” Another wrote, “I have no idea what it means so did not wanna give a biased answer.”

***Theme 2: Relationship Interpretations.*** Despite the unclear meaning of the items, many participants tried to rate them by drawing on information about their own romantic relationships. Across the sample, 88.89% of the participants referenced their own relationships in response to at least one item, and 23.46% of them referenced their relationship in response to all three items. Many of these responses (41.46%) drew on global, usually positive feelings about the relationship. For example, when asked to justify their rating of the *snowdrop* item, one participant wrote, “I love the person I am in a relationship with. 7 was the highest number I could choose to indicate how much I love them.” Some participants noted that although they did not know what the item meant, they nevertheless rated it positively because they felt positively about their relationship overall. For example, when asked why they rated the *umbra* item positively, one participant wrote, “Because this placeholder word feels like a positive word, and I appreciate the positive things my relationship brings.” Another wrote, “I wasn't sure what umbra meant. But I appreciate everything that comes with my relationship, so I clicked ‘agree.’”

Additionally, many of the responses (68.29%) drew on specific, concrete relationship qualities, often including a multitude of them simultaneously. For example, when asked what they were thinking about when completing the *snowdrop* item, one participant wrote, “I was thinking of the consistent support, affection, and small acts of kindness I receive in my relationship, which make me feel valued and appreciated.” Another wrote, “I thought of comforting moments. Where my partner was caring for me, giving me extra attention and comfort because I was feeling down.”

***Theme 3: Item Interpretations.*** Finally, many participants attempted to infer the meaning of the items based on the focal word within the item, usually drawing on its connotations or associations with other concepts. Across the sample, 74.07% of the participants attempted to interpret the focal word in at least one item, and 37.04% of them attempted to interpret the focal word for all three items. As with the relationship interpretations, some of the item responses were general or holistic (40.63%). For example, when explaining why they rated the *umbra* item positively, one participant wrote, “Because it sounds like a good word, it seems like it relates to the wholesomeness.” Another participant, who rated the *umbra* item negatively, wrote, “I know that umbra means shadow, so for me the statement had a negative connotation, and hence I disagreed with it as I am happy in my relationship.”

Other participants offered more specific interpretations in their responses (66.41%), often tying the item back to relationship concepts. For example, when asked to define the *reseda* item, one participant wrote, “I think this means to treasure the realness and naturalness of your relationship. Much like the flower a reseda is strong and green which is resembled through a relationship.” In response to the *snowdrop* item, one participant wrote, “it likely means finding joy, love, and beauty in the small, precious moments or gestures I receive from our relationship.” Another participant, in response to the *umbra* item, wrote, “To me umbra means natural, genuine, and wholehearted. I love my man and that’s exactly what our relationship is to me.”

**Study 2 Supplemental Materials**

**Participants**

Supplemental details regarding the participant demographics can be seen in Supplemental Table 5.

Supplemental Table 5: Detailed participant demographics for Study 2

|  | ***n* (%)** |
| --- | --- |
| **Gender/Sex** |  |
| Cisgender | 457 (95.8) |
| Transgender | 15 (3.1) |
| Intersex | 1 (0.2) |
| Prefer not to say | 4 (0.8) |
| **Gender** |  |
| Man | 149 (31.2) |
| Woman | 315 (66.0) |
| Non-binary | 6 (1.3) |
| Specify | 6 (1.3) |
| Prefer not to say | 1 (0.2) |
| **Sexual Orientation** |  |
| Heterosexual | 358 (75.1) |
| Gay or Lesbian | 18 (3.8) |
| Queer | 10 (2.1) |
| Bisexual | 72 (15.1) |
| Uncertain or questioning | 7 (1.5) |
| Specify | 7 (1.5) |
| Prefer not to say | 3 (0.6) |
| **Relationship Status** |  |
| Casually dating one person | 38 (8.0) |
| Seriously dating more than one person | 2 (0.4) |
| Seriously dating one person, but not living together | 266 (55.8) |
| Living with my partner | 102 (21.4) |
| Engaged | 26 (5.5) |
| Married | 43 (9.0) |
| **Ethnicity/Race*** |  |
| Aboriginal | 7 |
| Arab / West Asian | 21 |
| Black | 45 |
| Chinese | 60 |
| European | 43 |
| Filipino | 9 |
| Japanese | 3 |
| Korean | 6 |
| Latin American | 18 |
| South Asian | 34 |
| South East Asian | 18 |
| White/Caucasian | 264 |
| Specify | 8 |
| Prefer not to say | 1 |
| **Religion*** |  |
| Christian Protestant | 79 |
| Christian Catholic | 132 |
| Christian Orthodox | 17 |
| Jewish | 8 |
| Muslim | 21 |
| Sikh | 5 |
| Hindu | 16 |
| Buddhist | 10 |
| Atheist/Agnostic | 200 |
| Specify | 20 |

***Note****.* Participants responded to questions denoted (*****) with a select all that apply format rather than a single forced-choice format.

**Additional Analyses: Taking an SEM Approach**

In the models presented in the main text, we used regression-based approaches (linear regression and multilevel modelling) to provide evidence of Pseudo’s predictive and incremental validity. We chose this analytic approach because it is most commonly used in relationship science (Sakaluk et al., 2024). However, regression-based approaches are limited by their assumption of perfect measurement (Westfall & Yarkoni, 2016; Wang & Eastwick, 2020). Structural equation modelling (SEM) has been offered as a possible safeguard against the problems introduced by measurement unreliability, by explicitly modelling it, and thereby providing more calibrated regression slopes purged of any measurement error influence (Sakaluk et al., 2024; Wang & Eastwick, 2020). Thus, we also tested Pseudo’s incremental validity over and above relationship satisfaction using SEM.

We first used confirmatory factor analysis to test whether Pseudo is empirically distinguishable from relationship satisfaction. We entered the 10 Pseudo items and three satisfaction items into both two-factor and one-factor models, which we then compared. A model with the Pseudo and satisfaction items loading onto two respective factors fit the data well, RMSEA = .061, CFI = .977, SRMR = .030, with the two latent factors correlated at *r* = .611; see Supplemental Figure 1. In contrast, a model with all items loading onto the same factor fit the data poorly, RMSEA = .195, CFI = .763, SRMR = .096. The two-factor model fit significantly better than the one-factor model, ∆χ2 = 1361.70, *p* < .001.

*Supplemental Figure 1.* Pseudo and satisfaction represented as separate latent factors.

We also tested whether Pseudo predicted changes in relationship outcomes over time using SEM. In these models, we constructed three latent factors using: the 10 Pseudo items from Week 2, the three relationship outcome items at Week 4 (the last timepoint), and the same relationship items at Week 1 (the first timepoint), respectively. We then added paths to predict the Week 4 relationship outcome using the equivalent baseline relationship construct and Pseudo. Results are shown in Supplemental Table 6. Results closely mirrored those of the multiple regression models: latent Pseudo at Week 2 significantly predicted latent versions of each relationship outcome measure tested at Week 4, controlling for that measure at baseline (Week 1).

Finally, we tested these models with Week 2 treated as baseline instead of Week 1 (i.e., predicting change over three weeks instead of four; results not depicted). Here too, the results were highly similar to those of the multiple regression models. Latent Pseudo significantly predicted changes in trust, marginally predicted changes in appreciation, and did not predict changes in commitment or satisfaction over time. Overall, Pseudo significantly predicted changes in relationship outcomes in six out of eight multiple regression models tested, and five out of eight structural equation models tested.

Supplemental Table 6: Pseudo predicts change in relationship variables using SEM

| Predictor | Week 4 Relationship Outcome | | | Model Fit Indices | | |
| --- | --- | --- | --- | --- | --- | --- |
|  | β | *SE* | *p* | RMSEA | CFI | SRMR |
| ***Model 1*** |  |  |  |  |  |  |
| Pseudo | .162 | .073 | .001 | .053 | .976 | .034 |
| Week 1 Satisfaction | .654 | .132 | < .001 |  |  |  |
| ***Model 2*** |  |  |  |  |  |  |
| Pseudo | .186 | .085 | .003 | .049 | .980 | .029 |
| Week 1 Commitment | .577 | .167 | < .001 |  |  |  |
| ***Model 3*** |  |  |  |  |  |  |
| Pseudo | .260 | .087 | .003 | .068 | .954 | .049 |
| Week 1 Appreciation | .601 | .178 | < .001 |  |  |  |
| ***Model 4*** |  |  |  |  |  |  |
| Pseudo | .194 | .093 | .001 | .050 | .975 | .033 |
| Week 1 Trust | .663 | .206 | < .001 |  |  |  |

**Study 2 Supplemental Materials**

**Participants**

Supplemental details regarding the participant demographics can be seen in Supplemental Table 7.

Supplemental Table 7: Detailed participant demographics for Study 3

|  | ***n* (%)** |
| --- | --- |
| **Transgender** |  |
| No | 237 (94.0) |
| Yes | 11 (4.4) |
| Don’t know | 2 (0.8) |
| **Gender** |  |
| Man | 112 (44.4) |
| Woman | 130 (51.6) |
| Non-binary, genderqueer  Agender, genderless | 5 (2.0)  3 (1.2) |
| **Sexual Orientation** |  |
| Straight | 175 (69.4) |
| Gay | 5 (2.0) |
| Lesbian | 12 (4.8) |
| Queer | 4 (1.6) |
| Bisexual, pansexual | 51 (20.2) |
| Asexual  Other | 2 (0.8)  1 (0.4) |
| **Relationship Status** |  |
| Dating my current partner and others | 4 (1.6) |
| Dating my current partner exclusively | 119 (47.2) |
| Common-law  Civil partnership  Engaged  Married | 35 (13.9)  3 (1.2)  19 (7.5)  70 (27.8) |
| **Ethnicity/Race** |  |
| White, Caucasian, Anglo | 210 (83.3) |
| Black, African, Caribbean | 5 (2.0) |
| Hispanic, Latino/a, Chicano/a | 5 (2.0) |
| East Asian | 5 (2.0) |
| South Asian | 5 (2.0) |
| Southeast Asian | 7 (2.8) |
| Mixed or multiple ethnic groups | 9 (3.6) |
| Other | 4 (1.6) |
| **Education** |  |
| Primary education | 1 (0.4) |
| Lower secondary degree | 2 (0.8) |
| Upper secondary degree | 41 (16.3) |
| Vocational degree | 21 (8.3) |
| Undergraduate degree | 106 (42.1) |
| Master’s degree  Doctorate or professional degree  Other educational degree | 64 (25.4)  6 (2.4)  9 (3.6) |

Percentages may not add up to 100% because of missing data.

**Preregistration Deviations**

The Study 2 analysis plans were preregistered in two phases: cross-sectional analyses (available here), and longitudinal analyses (available here). There were some minor deviations from the preregistrations, summarized below:

1. For the cross-sectional analyses: one preregistered model included a variable (love) that was not actually measured in the study. We replaced that variable with “satisfaction” in the relevant model.
2. For the longitudinal analyses, residual covariances across time were accidentally omitted from our preregistered analytic scripts (thankfully, this omission was caught and remedied by an observant reviewer). Adding these covariances did not change the pattern of results.
3. For the sake of page space, we were unable to report all preregistered models in the main text. Any preregistered model not included in the main text is instead reported below (i.e., in the supplement).
4. Some additional analyses were tested that were not preregistered, both in the main text and in the supplemental file. These analyses are clearly noted as exploratory, as opposed to confirmatory.

**Results**

**Analytic Strategy**

As described briefly in the main text, we tested associations of interest with a series of preregistered dyadic SEM analyses with latent variables (Sakaluk et al., 2024). The data were structured in dyadic format (one row per couple), and the dyads were treated as distinguishable, with the actor and partner roles assigned randomly for each couple. All models were fit using the lavaan package (Rosseel, 2012) in *R* (R Core Team, 2023), via robust maximum likelihood estimation when all item responses had more than four response categories, and via a robust categorical estimator when item responses contained four or fewer response categories (i.e., models with depression or general anxiety measures)(Rhemtulla et al., 2012). All models were identified and their scales were set using a fixed-factor approach, whereby latent variances were constrained to 1, and all factor loadings were estimated (Little, 2013). Dependency within the measurement model was addressed in two ways, depending on the nature of our analyses (and the corresponding data). For cross-sectional models, residual variances of indicator variables were allowed to covary between partners (e.g., estimating the residual covariance between the first Pseudo item for both partners) (see Sakaluk et al., 2021). For longitudinal models, meanwhile, residual variances were allowed to covary for the same item both between-partner (as in the cross-sectional models; Sakaluk et al., 2021), and also across time (e.g., estimating the residual covariance between the first Pseudo item at the first and second timepoint, first and third timepoint, and second and third timepoint; Little, 2013).

As our analyses relied on (with the exception of Pseudo) measures with long-standing histories of psychometric evaluation and validation, we were less interested in appraisals of model fit in a general sense—which might otherwise pick up on measurement discrepancies in our particular sample—and instead focused on nested model comparisons of model fit, for estimated patterns of association with Pseudo versus models constrained to our preregistered hypothesized thresholds.

**Evaluative Versus Semi-Evaluative Relationship Measures (Confirmatory)**

As reported in the main text, Pseudo was strongly associated with other relationship measures, and weakly associated with non-relationship measures. We also examined whether Pseudo was particularly associated with measures that tap into global relationship sentiments (e.g., satisfaction, commitment), compared to relationship measures that are arguably less evaluative (e.g., attachment). Specifically, we conducted a series of preregistered models pairing one more evaluative construct with one less evaluative construct.

In each model, Pseudo items, items from a more evaluative measure (e.g., trust), and items from a less evaluative measure (e.g., attachment anxiety) were all loaded onto separate factors. We preregistered that if the correlation between Pseudo and the more evaluative measure was stronger than the correlation between Pseudo and the less evaluative measure, that would be taken as evidence that Pseudo is tapping into biases that are specifically sentiment-oriented. To test this hypothesis, we used nested model comparisons to compare an unconstrained version of each model (correlations freely estimated) to a model in which correlations between latent Pseudo and each other factor were constrained to equal. If Pseudo tracks evaluative measures to a greater extent, we anticipated worse fit when correlations were constrained to equality, with Pseudo’s correlation in the unconstrained model being higher in magnitude to the evaluative measure than the semi-evaluative measure. Note that for these models, negatively-valenced items (e.g., attachment avoidance) were recoded to be in the positive direction, in order to ensure shared directionality between the competing correlations among Pseudo and the target evaluative and semi-evaluative measures of comparison, which were necessary for the interpretability of the nested-model comparison.

Results can be seen in Supplemental Table 8. Indeed, nearly all correlations between Pseudo and evaluative measures were descriptively higher than the less-evaluative measures. However, the constrained models resulted in significantly worse fit for only four of the nine models tested. The evaluative relationship constructs (e.g., satisfaction, commitment, closeness, trust) tended to be moderately correlated with Pseudo, with a median *r* of .434 (*range* = .234 to .468). However, there was varying strength in the associations between Pseudo and the ostensibly less-evaluative constructs. Attachment anxiety was weakly associated with Pseudo (*range* = .013 to .121), and all three models that used attachment anxiety as the less evaluative measure shows support for our hypothesis: Pseudo was significantly more strongly associated with satisfaction, trust, and responsiveness than it was with attachment anxiety. However, the other two less evaluative constructs (attachment avoidance and relationship mindfulness) were moderately correlated with Pseudo (*range* = .239 to .434). Models using either of these measures as the less evaluative construct did not support the hypothesis, with one exception (Pseudo was significantly more strongly associated with satisfaction than it was with attachment avoidance). Overall, results provided tentative, mixed support for our hypothesis.

Supplemental Table 8

*How Strongly Is Pseudo Associated with More Versus Less Evaluative Relationship Constructs?*

|  | Relationship Measures to Compare | | Correlations Between Pseudo and Each Other Factor (Actor, Partner) | | Comparing Constrained Vs. Unconstrained Models | | |
| --- | --- | --- | --- | --- | --- | --- | --- |
| Preregistered? | More Evaluative | Less Evaluative | More Evaluative | Less Evaluative | Δχ² | *df diff.* | *p* |
| Yes | Satisfaction | Attachment Anxiety | .451, .451 | .025, .121 | 15.266 | 2 | < .001 |
| Yes | Commitment | Attachment Avoidance | .325, .378 | .239, .420 | .912 | 2 | .634 |
| Yes | Closeness | Relationship Mindfulness | .435, .457 | .251, .428 | 2.96 | 2 | .228^a^ |
| Yes | Trust | Attachment Anxiety | .382, .234 | .013, .113 | 6.290 | 2 | .043 |
| Yes | Positive Relationship Evaluations | Attachment Avoidance | .406, .468 | .246, .424 | 2.742 | 2 | .254 |
| Yes | Negative Relationship Evaluations | Relationship Mindfulness | .347, .287 | .262, .422 | 1.634 | 2 | .442^a^ |
| Yes | Responsiveness | Attachment Anxiety | .337, .433 | .022, .121 | 9.349 | 2 | .009 |
| No | Satisfaction | Attachment Avoidance | .450, .452 | .240, .422 | 6.08 | 2 | .048 |
| No | Satisfaction | Relationship Mindfulness | .451, .454 | .263, .434 | 3.25 | 2 | .197^a^ |

*Note.* In the constrained models, correlations between the Pseudo factor and the more versus less evaluative relationship factors were constrained to equal.

^a^These models generated a warning that the variance-covariance matrix of parameter estimates were not positive definite. We inspected these matrixes manually, and confirmed that the values in question were exceptionally close to zero. This suggests that the warning is caused by the relatively small sample size, rather than a bona fide identification issue. As these models followed the same general pattern of specification as others in this table (which did not have identification issues) we therefore proceeded with their interpretation.

**Associations Between Pseudo and Partner Reports (Confirmatory)**

We also conducted preregistered models testing how one partner’s Pseudo is associated with the other partner’s relationship quality. If Pseudo is capturing something dyadic, such as true relationship quality, we would expect an association akin to those found with relationship partner reports (e.g., the link between one partner’s satisfaction and the other partner’s commitment). In contrast, if Pseudo is primarily capturing person-specific biases, we would expect an association akin to those found with non-relational partner reports (e.g., the link between one partner’s satisfaction and the other partner’s agreeableness). We tested this with a series of confirmatory models probing the association between one partner’s Pseudo and the other partner’s relationship quality.

We specified correlated factor models in which Pseudo and relationship quality items were loaded onto separate factors. We preregistered that if the correlation between one partner’s Pseudo and the other partner’s relationship quality was higher than .2 (the average inter-partner correlation between BFI personality dimensions according to Gattis et al., 2004), that would be taken as evidence that Pseudo is capturing real dyadic effects. We used nested model comparisons to compare an unconstrained version of each model (correlations freely estimated) to a model in which correlations between one partner’s latent Pseudo and the other partner’s latent relationship quality were constrained to .2.

Results can be seen in Supplemental Table 9. Constraining the inter-partner correlations to .2 resulted in marginally worse fit for only two of the seven tested models, and only because one or more associations in those models were *weaker* than .20. These results therefore suggest that Pseudo is likely capturing intraindividual, rather than dyad-level phenomena.

Supplemental Table 9

*How Strongly Is One Partner’s Pseudo Associated with the Other Partner’s Relationship Variables?*

| Measure | Inter-Partner Correlations | | Comparing Constrained Vs. Unconstrained Models | | |
| --- | --- | --- | --- | --- | --- |
|  | Minimum | Maximum | Δχ² | *df diff.* | *p* |
| Satisfaction | .149 | .201 | .265 | 2 | .876 |
| Commitment | .139 | .156 | .562 | 2 | .755 |
| Trust | -.029 | .096 | 5.066 | 2 | .079 |
| Closeness | .089 | .139 | 1.344 | 2 | .511 |
| Responsiveness | .182 | .330 | 1.952 | 2 | .377 |
| Positive Relationship Evaluations | .073 | .181 | 1.895 | 2 | .388 |
| Negative Relationship Evaluations | -.255 | .020 | 5.453 | 2 | .065 |

*Note.* In the constrained models, correlations between one partner’s Pseudo factor and the other partner’s relationship quality factor were constrained to .2.

**Stability of Pseudo, Satisfaction, and Commitment Over Time**

In the main text, we report on latent models testing whether constructs of interest (Pseudo, satisfaction, and commitment) vary significantly over time. At the request of a reviewer, we also examined the latent stabilities between each timepoint for each variable; see Supplemental Figure 2. Pseudo’s stability between timepoints (M = .636, SD = .096, range = .63 to .84) was similar to commitment (M = .673, SD = .079, range = .72 to .90), and satisfaction (M = .669, SD = .03, range = .74 to .82).

*Supplemental Figure 2.* Latent stabilities for Pseudo, Satisfaction, and Commitment.

**Stability of the Association Between Pseudo and Satisfaction**

We conducted preregistered models examining the stability of Pseudo’s association with satisfaction over time. We tested a model in which latent satisfaction factors at each time point were regressed onto a latent baseline Pseudo factor (confirmatory). In the first version of the model, all pathways between satisfaction and Pseudo were freely estimated. We compared this to a model in which the pathways were constrained to be equal. We preregistered that if the pathways between baseline Pseudo and later satisfaction timepoints were weaker than the pathway with baseline satisfaction, and if the constrained model has significantly worse fit, that would be taken as evidence that the association between Pseudo and satisfaction weakens over time. For comparison, we conducted a similar pair of models in which Pseudo was replaced with commitment (exploratory).

Results can be seen in Supplemental Table 10. Satisfaction was strongly associated with Pseudo when both constructs were measured at the same timepoint, but that association was much weaker when satisfaction was measured at a later timepoint. A nested model comparison test revealed that constraining the paths between Pseudo and satisfaction at each wave to be equal resulted in a significantly worse-fitting model compared to when the paths were freely estimated. These results suggests that the association between Pseudo and satisfaction is time-specific, in that it is strongest when both constructs are measured at the same timepoint. In contrast, we did not find the significant results when Pseudo was replaced with the commitment. In other words, commitment’s association with relationship satisfaction appears to be stable across time in a way that Pseudo’s association with satisfaction is not.

Supplemental Table 10

*How Stable Are Associations Between Constructs Over Time?*

| Measures | Pathways For Satisfaction at Each Wave | | | Comparing Constrained Vs. Unconstrained Models | | |
| --- | --- | --- | --- | --- | --- | --- |
|  | Baseline | Wave 4 | Wave 5 | Δχ² | *df diff.* | *p* |
| Baseline Pseudo Versus Satisfaction at Each Wave | .402, .426 | -.095,  -.087 | .068, .136 | 28.972 | 4 | < .001 |
| Baseline Commitment Versus Satisfaction at Each Wave | .325, .464 | .355,  .182 | .527, .627 | 7.055 | 4 | .133 |

*Note.* In the constrained models, associations between latent satisfaction at each wave and the latent baseline construct were constrained to equal.

**Measurement Models (Confirmatory)**

Our initial, preregistered analytic strategy indulged two possible structures by which Pseudo’s confounding force might pervade analyses: a correlated factor model (in which Pseudo items load exclusively onto a Pseudo factor, which is then correlated with other factors in a model) and a bifactor model (in which items from other measures cross-load onto a generalized Pseudo factor, along with Pseudo items; see Morin et al., 2016). In comparing these divergent accounts of Pseudo, we found tentative support for the correlated factor model over the bifactor model. For space concerns, we reported only the key correlated factor results in the main text. All preregistered bifactor analyses are reported below. They largely support the same pattern of substantive results as the correlated factor models.

***Associations Between Pseudo and Other Relationship Measures.*** We conducted a series of bifactor models, each with two latent factors: a construct-specific factor comprised only of the items from the relevant relationship measure, and a bifactor with loadings from both Pseudo and the relationship measure. We preregistered that if relationship measure items tended to load strongly (a standardized loading of .40 or larger) onto the bifactor, that would be taken as evidence that Pseudo is contaminating the item-level variance of those relationship measures. To test this hypothesis, we used nested model comparisons to compare an unconstrained version of each model (loadings freely estimated) to a model in which bifactor loadings for the relationship items were constrained to .4 (or -.4 for negatively valenced items).

Results can be seen in Supplemental Table 11. Consistent, appreciable loadings onto the Pseudo bifactor emerged for five out of ten of the tested relationship measures (satisfaction, commitment, closeness, responsiveness, and positive relationship evaluations). Constraining the bifactor loadings to .4 resulted in significantly worse fit for four constructs, and marginally worse fit for two constructs. Overall, we find mixed support for a bifactor structure between Pseudo and other relationship measures, with better support for measures that are more evaluative (e.g., satisfaction, commitment), and less support for measures that are less evaluative and more trait-like in nature (e.g., attachment style).

Supplemental Table 11

*How Strongly Is Pseudo Associated with Relationship Constructs at the Measurement Level?*

| Measure | Range of Standardized Loadings | | Comparing Constrained Vs. Unconstrained Models | | |
| --- | --- | --- | --- | --- | --- |
|  | Minimum | Maximum | Δχ² | *df diff.* | *p* |
| Satisfaction | .374 | .465 | 9.572 | 6 | .144 |
| Commitment | .299 | .413 | 7.472 | 6 | .279 |
| Trust | .199 | .327 | 17.952 | 6 | .006 |
| Closeness | .335 | .472 | 12.304 | 6 | .056 |
| Responsiveness | .245 | .364 | 2.984 | 8 | .935 |
| Positive Relationship Evaluations | .277 | .474 | 12.12 | 8 | .146 |
| Negative Relationship Evaluations | -.308 | -.125 | 35.833 | 8 | < .001 |
| Attachment Anxiety | -.067 | .194 | 18.896 | 12 | .091 |
| Attachment Avoidance | -.412 | -.032 | 25.928 | 12 | .011 |
| Relationship Mindfulness | -.047 | .357 | 66.158 | 30 | < .001 |

*Note.* In the constrained models, loadings of relationship items onto the Pseudo bifactor were constrained to .4.

***Evaluative Versus Semi-Evaluative Relationship Measures.*** We next examined how strongly Pseudo is associated with more versus less evaluative relationship constructs. We again conducted a series of bifactor models, each with three latent factors: two construct-specific factor comprised of the items from the more versus less evaluative relationship measure, respectively, and a bifactor with loadings from Pseudo and both relationship measures. We preregistered that if the more evaluative relationship items loaded more strongly onto the bifactor than the less evaluative items, that would be taken as evidence that Pseudo reflects a sentiment-oriented item-level contaminant (as opposed to some other relational bias). To test this hypothesis, we used nested model comparisons to compare an unconstrained version of each model (loadings freely estimated) to a model in which bifactor loadings for the more versus less evaluative items were constrained to be equal. As above, negatively valenced items were recoded to be in the positive direction for these models.

Results can be seen in Supplemental Table 12. Loadings onto the Pseudo bifactor tended to be moderate for the more evaluative relationship measures, and weak for the less evaluative relationship measures. Constraining the bifactor loadings for more versus less evaluative items to equal resulted in significantly worse fit for four pairs of constructs, and marginally worse fit for one pair of constructs. Overall, we find mixed support for the hypothesis that Pseudo is particularly associated with more versus less evaluative relationship constructs at the measurement level.

Supplemental Table 12

*How Strongly Is Pseudo Associated with More Versus Less Evaluative Relationship Constructs at the Measurement Level?*

|  | Relationship Measures to Compare | | Range of Standardized Loadings | | Comparing Constrained Vs. Unconstrained Models | | |
| --- | --- | --- | --- | --- | --- | --- | --- |
| Preregistered? | More Evaluative | Less Evaluative | More Evaluative | Less Evaluative | Δχ² | *df diff.* | *p* |
| Yes | Satisfaction | Attachment Anxiety | .369, .424 | .051, .117 | 6.005 | 2 | .0497 |
| Yes | Commitment | Attachment Avoidance | .297, .415 | .117, .381 | 2.443 | 2 | .295 |
| Yes | Closeness | Relationship Mindfulness | .261, .462 | .088, .197 | 8.212 | 2 | .016 |
| Yes | Trust | Attachment Anxiety | .204 | .101, .206 | .473 | 2 | .790^a^ |
| Yes | Positive Relationship Evaluations | Attachment Avoidance | .274, .456 | .120, .379 | 2.357 | 2 | .308 |
| Yes | Negative Relationship Evaluations | Relationship Mindfulness | .159, .238 | .080, .183 | .111 | 2 | .946^b^ |
| Yes | Responsiveness | Attachment Anxiety | .252, .350 | .057, .120 | 67.023 | 2 | < .001 |
| No | Satisfaction | Attachment Avoidance | .390, .487 | .121, .367 | 5.445 | 2 | .066 |
| No | Satisfaction | Relationship Mindfulness | .404, .430 | .093, .194 | 23.047 | 2 | < .001^b^ |

*Note.* In the constrained models, loadings of more versus less evaluative items onto the Pseudo bifactor were constrained to be equal.

***Associations Between Pseudo and Non-Relationship Measures.*** We next examined how strongly Pseudo is associated with relational versus non-relational constructs at the measurement level. We conducted a series of bifactor models, each with three latent factors: two construct-specific factors comprised of the items from the relational versus non-relational measures, respectively, and a bifactor with loadings from Pseudo and both other measures. We preregistered that if the relational items loaded more strongly onto the bifactor than the less relational items, that would be taken as evidence that Pseudo reflects a relationship-oriented item-level contaminant (as opposed to a more domain-general bias).

Results can be seen in Supplemental Table 13. As with the structural models, our hypothesis was generally supported at the measurement level, with the constrained model resulting in significantly worse fit in seven out of nine models tested. Consistent with the results of the other models, loadings onto the Pseudo bifactor tended to be moderate for the evaluative relationship measures, and weaker for the less evaluative and non-relational measures. Constraining the relational versus non-relational bifactor loadings to equal generally resulted in worse model fit.

Supplemental Table 13

*How Strongly Is Pseudo Associated with Relational Versus Non-Relational Constructs at the Measurement Level?*

|  | Measures to Compare | | Range of Standardized Loadings | | Comparing Constrained Vs. Unconstrained Models | | |
| --- | --- | --- | --- | --- | --- | --- | --- |
| Preregistered? | Relational | Non-Relational | Relational | Non-Relational | Δχ² | *df diff.* | *p* |
| Yes | Satisfaction | Satisfaction with Life | .406, .434 | .095, .148 | 10.617 | 2 | .005 |
| Yes | Closeness | Meaning in Life | .260, .461 | .071, .128 | 8.887 | 2 | .012 |
| Yes | Positive Relationship Evaluations | Life Satisfaction | .281, .399 | .095, .148 | 3.93 | 2 | .140 |
| Yes | Negative Relationship Evaluations | Depression | -.290, -.168 | -.095, .026 | 12.431 | 2 | .002^a^ |
| Yes | Attachment Anxiety | Generalized Anxiety | .198, .471 | .145, .485 | 13.230 | 2 | .001^a^ |
| Yes | Attachment Anxiety | Neuroticism | .058, .137 | .065, .201 | .269 | 2 | .874 |
| Yes | Attachment Avoidance | Agreeableness | .161, .410 | .011, .342 | 12.445 | 2 | .002^b^ |
| Yes | Relationship Mindfulness | General Mindfulness | .065, .219 | -.122, .202 | 31.526 | 7 | < .001^c^ |
| No | Satisfaction | Meaning in Life | .404, .419 | .075, .142 | 18.788 | 2 | < .001 |

*Note.* In the constrained models, correlations between the Pseudo factor and the relational versus non-relational factors were constrained to equal.

***Associations Between Pseudo and Partner Reports.*** We next examined associations between one partner’s Pseudo and the other partner’s relationship quality at the measurement level. We conducted bifactor models in which the relationship quality items were loaded onto a relationship factor, and items from both the relationship quality measure and Pseudo were loaded onto a Pseudo bifactor. We tested constrained versions of each model in which the associations between one partner’s relationship factor and the other partner’s latent bifactor were constrained to .20. Results can be seen in Supplemental Table 14. As with the structural models, the hypothesis was supported at the measurement level. Correlations between one partner’s relationship factor and the other partner’s Pseudo bifactor were generally smaller than .20. Constraining the correlations to .20 only resulted in significantly worse fit for models that had *smaller* correlations, rather than larger. These findings do not support the idea that Pseudo is tapping into true dyadic processes at the measurement level.

Supplemental Table 14

*How Strongly Is One Partner’s Pseudo Associated with the Other Partner’s Relationship Quality at the Measurement Level?*

| Measure | Cross-Partner Correlations | | Comparing Constrained Vs. Unconstrained Models | | |
| --- | --- | --- | --- | --- | --- |
|  | Minimum | Maximum | Δχ² | *df diff.* | *p* |
| Satisfaction | .093 | .145 | 1.375 | 2 | .503 |
| Commitment | .091 | .114 | 2.055 | 2 | .358 |
| Trust | -.048 | .066 | 7.4005 | 2 | .02 |
| Closeness | .014 | .049 | 5.216 | 2 | .074 |
| Responsiveness | .133 | .278 | 1.130 | 2 | .568 |
| Positive Relationship Evaluations | .006 | .111 | 4.886 | 2 | .087 |
| Negative Relationship Evaluations | -.210 | .091 | 6.54 | 2 | .038 |

*Note.* In the constrained models, loadings of relationship items onto the Pseudo bifactor were constrained to .4.

**Additional Analytic Notes**

***Experimental Intervention.*** The Study 2 longitudinal data were collected in the context of an unrelated experimental intervention. We therefore tested measurement invariance of Pseudo and structural equivalence across the intervention condition (n = 65) versus the control condition (n = 61) at each of the three timepoints (Little, 2013). At the measurement mode level, we failed to detect any significant noninvariance between the groups in factor loadings, Δχ²(30) = 32.58, p =.34, or item intercepts, Δχ²(24) = 20.11, p =.69. Likewise, at the structural level, we failed to detect any differences in latent variances or covariances, Δχ²(15) = 16.57, p =.35. There was some weak evidence of latent mean differences between groups, Δχ²(6) = 16.76, p =.01. However, we were not concerned by these given that our hypotheses about Pseudo were about its associations rather than central tendency (see Little, 2013). In sum, results suggest that Pseudo’s measurement model and associative nature were unaffected by the experimental design under which data were collected.

***Gamma matrix warnings.*** Some of the preregistered models generated a warning that the sample was too small to compute the gamma matrix. This occurred because those models required a categorical estimator, for which full-information maximum likelihood treatment of missingness is not available (and so 10 cases with missing data were excluded). However, the gamma matrix is only necessary to inform more specialized tests of CFA models, and therefore, its omission did not affect our process of model appraisal or comparison.

***Heywood cases.*** Some models generated Heywood cases due to the loadings for one of the variables being so close to 1 (fully standardized) that a viable non-negative value could not be found via maximum likelihood estimation. As a solution, we constrained those loadings to equality across partners (along with the corresponding residual variance).

***Not positive definite.*** Some models generated a warning that the variance-covariance matrix of parameter estimates was not positive definite. We inspected these matrixes manually and confirmed that the values in question were exceptionally close to zero.
